# Tuning *FLO1* Expression via Promoter Engineering Modulates Flocculation Degree and Acetic Acid Stress Tolerance in *Saccharomyces cerevisiae*

**DOI:** 10.3390/jof12010047

**Published:** 2026-01-09

**Authors:** Pei-Liang Ye, Wei-Bin Wang, Liang Xiong, Guang-Xian Peng, Cheng Cheng, Xin-Qing Zhao

**Affiliations:** 1 State Key Laboratory of Microbial Metabolism, School of Life Sciences and Biotechnology, Shanghai Jiao Tong University, Shanghai 200240, China; 2 School of Biotechnology and Food Engineering, Hefei Normal University, Hefei 230601, China

**Keywords:** *Saccharomyces cerevisiae*, cell flocculation, acetic acid stress, ethanol fermentation, promoter engineering

## Abstract

Robust yeast tolerance to inhibitors is essential for lignocellulosic biorefinery. Although cell flocculation is known to enhance acetic acid stress tolerance, the impact of its intensity remains unclear. In this study, engineered *S. cerevisiae* strains with distinct floc sizes were constructed through promoter engineering. The native *FLO1* promoter in the non-flocculating laboratory strain BY4741 was replaced with either the constitutive strong promoter *PGK1p* or the ethanol-inducible promoter *TPS1p* using CRISPR-Cas9-mediated genome editing, resulting in strongly and moderately flocculating strains BY4741 *PGK1p-FLO1* and BY4741 *TPS1p-FLO1*, respectively. It was revealed that the BY4741 *PGK1p-FLO1* showed a survival advantage in the late-stage fermentation and severe stress condition in the presence of 7.5 g/L acetic acid, while BY4741 *TPS1p-FLO1* exhibited superior growth and fermentation performance under 5.0 g/L acetic acid stress. Further studies suggested that the enhanced acetic acid tolerance in flocculating cells was associated with their ability to maintain significantly higher intracellular ATP levels under stress. Our work highlights the importance of optimizing flocculation properties for robust industrial fermentation, and also provides a strategic basis for engineering stress-tolerant yeast strains for efficient fermentation in inhibitor-rich cellulosic hydrolysates.

## 1. Introduction

The global transition towards sustainable energy and chemical production has intensified the development of lignocellulosic biorefineries, which utilize renewable raw materials such as agricultural and forestry residues [1]. Through pretreatment and enzymatic hydrolysis, various sugars such as glucose, xylose, and arabinose can be generated from lignocellulose. These sugars serve as feedstocks for microbial fermentation to manufacture a series of products [2,3]. However, a major challenge in the efficient bioconversion of lignocellulosic hydrolysates is the presence of inhibitors released during the pretreatment process. One of the most common inhibitors is acetic acid, which originates from the deacetylation of hemicellulose [4]. High concentration of acetic acid impairs cellular functions by causing intracellular acidification, energy depletion, and oxidative stress, ultimately leading to reduced microbial growth and fermentation performance [5]. Therefore, improved tolerance towards acetic acid is crucial for the sustainable production of biofuels and biochemicals from lignocellulosic biomass.

Various strategies, including evolutionary engineering, genetic modification, and process optimization, have been explored to mitigate the negative effects of acetic acid on yeast cells [5,6,7]. As reported, supplementation of Zn^2+^ at a suitable concentration was conducive to increased growth ability and enhanced ethanol fermentation of yeast cells under the stress of acetic acid [8]. Manipulating the expression of key genes is also an effective approach to promoting acetic acid tolerance. For instance, previous studies have demonstrated that manipulation of genes involved in chromatin remodeling, histone modification as well as of the mitogen-activated protein kinase Hog1, endowed yeast strains with altered acetic acid resistance [9,10,11,12]. In addition, we also found that yeast cell flocculation affects stress tolerance. Flocculating yeast strains are particularly suitable for industrial ethanol production for its advantages in high-cell-density and continuous fermentation, cost-effective biomass separation and enhanced tolerance to multiple stresses [13,14]. We confirmed that flocculation conferred yeast cells with significantly improved tolerance to acetic acid and exhibited faster ethanol fermentation in lignocellulose hydrolysate [15]. Therefore, the construction of flocculating yeast is an efficient strategy for industrial biotechnology. Despite these advantages, excessive flocculation can result in the formation of large, dense cellular aggregates that hinder mass transfer, thereby restricting the diffusion of nutrients and oxygen to the cells within the core of the flocs [13]. This frequently leads to reduced growth rates, extended fermentation cycles, and compromised productivity. It was found that the size of floc particles affects ethanol tolerance, cell viability, and fermentation performance [16,17]. Furthermore, strong flocculation results in slower glucose consumption under non-stress conditions [15]. Consequently, achieving a balance between flocculation strength and metabolic activity is critical for maximizing fermentation efficiency, particularly in inhibitor-rich environments such as lignocellulosic hydrolysates. However, so far artificially modulated flocculation has not yet been achieved to engineer yeast stress tolerance and improve fermentation performance.

In yeasts, flocculation is a calcium-dependent, reversible process involving the cell aggregation mediated by various *FLO* genes, e.g., *FLO1*, *FLO5*, *FLO9*, *FLO10*, *FLO11*, *Lg-FLO*, *FLONL* and *FLON*, which exhibit significant size polymorphism across various yeast strains [14,18]. Among these, the *FLO1* gene is a key determinant of the flocculation phenotype. It has been reported that the heterologous expression of the *FLO1* gene from *S. cerevisiae* in *Komagataella phaffii* conferred both a flocculation phenotype and higher tolerance to D-lactic acid [18]. Our group has developed a self-flocculating yeast strain, SPSC01, which exhibits strong flocculation due to the expression of the *FLO1* gene [19]. In the laboratory yeast strain BY4741, transcription of the *FLO1* gene is re-pressed by the Tup1-Cyc8 co-repressor complex. Furthermore, a nonsense mutation in the transcription factor Flo8 impairs *FLO1* expression, resulting in a non-flocculating phenotype [20,21]. Therefore, engineering *FLO1* gene expression to modulate a proper flocculation degree represents a viable approach to investigating the relationship between flocculation and stress tolerance. Recent advances in synthetic biology and promoter engineering have enabled the fine-tuning of gene expression, offering a viable strategy for controlling flocculation. However, related studies on controlled flocculation under acetic acid stress have not been reported. In this study, we developed yeast strains exhibiting strong and moderate flocculation phenotypes by replacing the native *FLO1* promoter with constitutive or ethanol-inducible promoters in the non-flocculating laboratory strain *S. cerevisiae* BY4741, respectively. The aim of this study is to systematically characterize the different flocculation properties of the engineered yeast strains and to assess the influence of the degree of flocculation on their acetic acid tolerance and ethanol fermentation performance. The underlying molecular and physiological mechanisms responsible for flocculation-mediated stress protection were further investigated. Our results emphasized the importance of suitable flocculation degree in industrial fermentation, as well as the benefits of constructing efficient yeast strains for the conversion of lignocellulosic biomass.

## 2. Materials and Methods

### 2.1. Strains and Culture Media

All the yeast strains and plasmids employed in this study are listed in Table 1. *Escherichia coli* DH5α was cultivated in Luria–Bertani (LB) medium containing 5 g/L yeast extract, 10 g/L tryptone, and 10 g/L NaCl. LB medium supplemented with 100 μg/mL ampicillin was adopted for transformants selection and plasmid propagation. The host strain BY4741 and its engineered derivatives BY4741 *PGK1p-FLO1*, BY4741 *TPS1p-FLO1* were cultured in YPD medium (10 g/L yeast extract, 20 g/L peptone, 20 g/L glucose). Solid plates were prepared by adding 20 g/L agar to YPD medium. The culture medium was supplemented with 300 μg/mL G418 or 500 μg/mL hygromycin B for screening of the yeast transformants. For ethanol fermentation, YPD100 (100 g/L glucose, 4 g/L yeast extract, and 3 g/L peptone) with or without acetic acid supplementation, served as the fermentation medium.

### 2.2. Construction of Recombinant Yeast Strains

The recombinant yeast strains BY4741 *PGK1p-FLO1* and BY4741 *TPS1p-FLO1* were constructed by replacing the native *FLO1* promoter in BY4741 with the *PGK1* and *TPS1* promoters, respectively, using CRISPR-Cas9-based genome-editing technology. All primers used for strain construction are listed in Table S1. Firstly, the plasmid Cas9-G418 was transferred into the cells of BY4741 to generate strain BY4741-Cas9. Specific gRNA protospacer sequences targeting the *FLO1* promoter region were then fused to the gRNA scaffold using ClonExpress^®^ Ultra One Step Cloning Kit (Vazyme, Nanjing, China). Subsequently, both the gRNA plasmids and the corresponding linear donor DNA fragments were co-transformed into BY4741-Cas9. Positive transformants were selected on YPD agar plates supplemented with antibiotics, and then verified by PCR analysis followed by DNA sequencing.

### 2.3. Ethanol Fermentation

The fermentation performance of the yeast strains was evaluated in both flasks and bioreactors. Prior to ethanol fermentation, yeast cells stored at −80 °C were firstly inoculated into YPD medium and incubated for 12 h. This culture was then subcultured into fresh YPD medium for an additional 12 h. Subsequently, the seed culture was inoculated into fermentation medium to an initial optical density at 600 nm (OD_600_) of 0.2. To simulate a high-glucose cellulosic hydrolysate [22], batch fermentation was conducted in 250 mL flasks containing 100 mL fermentation medium which was composed of 4 g/L yeast extract, 3 g/L peptone and 100 g/L glucose. Fermentations were performed at 30 °C and 150 rpm under microaerobic conditions without pH control. To assess acetic acid tolerance, 5.0 g/L acetic acid was supplemented in the fermentation medium and the condition without external addition of acetic acid served as a control. Ethanol fermentation in a bioreactor (KF-2.5L, KoBio Tech, Seoul, Republic of Korea) with 1000 mL fermentation medium was performed under the condition of 30 °C, 150 rpm and 0.04 vvm aeration. When testing 7.5 g/L acetic acid stress, the pH was maintained at 4.0 throughout fermentation by controlled addition of sterilized 1.0 M NaOH solution. Fermentation samples were taken every 12 h, and the residual glucose and ethanol concentrations were quantified using high-performance liquid chromatography (HPLC, Waters Alliance e2695 HPLC, Waters, Milford, MA, USA).

### 2.4. Characterizations of Flocculated Particle

Scanning electron microscopy (SEM) observations were employed to analyze flocculation state of yeast cells. For sample preparation, cells in logarithmic growth phase were harvested, washed twice with PBS buffer, and fixed overnight in 2.5% glutaraldehyde (*v*/*v*) at 4 °C. After washing in PBS buffer for three times, samples were dehydrated with series of ethanol gradients: 50%, 70%, 90%, 95%, respectively. In every gradient, cells were incubated for 15 min, and then collected by centrifugation at 3000× *g* for 2 min. Critical point drying was then performed using liquid CO_2_. Subsequently, dried samples were evenly spread on the copper tape of the sample stage with toothpicks. After gold sputtering, the samples were observed using a scanning electron microscope (S-3400N, Hitachi, Tokyo, Japan).

Particle size distribution was quantified using a dynamic laser particle size analyzer (Mettler Toledo Particle Track G400, Greifensee, Switzerland). The instrument was calibrated with deionized water prior to analysis. For each measurement, the entire flask contents were transferred to the instrument-specific sample cup. Macro Mode with stirring at 150 rpm was utilized to measure the particle diameters, and data were recorded after 5 min of equilibration to ensure consistent dispersion.

### 2.5. Flocculation Rate Measurement

Yeast strains were subcultured twice in YPD medium to mid exponential phase, and then cells were collected by centrifugation at 3000× *g* for 2 min. After that, the yeast cells were washed twice with 0.1 M sodium citrate buffer (pH 4.5) to induce deflocculation. Flocculation rate was determined by optical density assay as follows: Initial OD_600_ value was adjusted to about 2.0 with 0.1 M sodium citrate buffer (pH 4.5), and the accurate optical density was defined as A. 5 mL cell suspension was centrifuged and washed twice with flocculant solution which contained 50 mM NaAc and 1 g/L CaCl_2_ (pH 4.5) to re-flocculation. After vortexing at maximum speed for 30 s, the test tubes were stilled for 5 min totally. 200 μL supernatant was taken immediately for OD_600_ detection using a Multiskan GO 1510 microplate reader (Thermo Fisher Scientific, Waltham, MA, USA), and the optical density was defined as B. Flocculation rate was calculated by the equation C = (1 − B/A) × 100% [23].

### 2.6. Real-Time Quantitative PCR Analysis

Yeast cells cultivated in fermentation medium (YPD100) supplemented with 5 g/L acetic acid were harvested at logarithmic growth phase. Total RNA was extracted using a HiPure Yeast RNA Kit (Magen Biotechnology, Guangzhou, China) following the manufacturer’s instructions. For cDNA synthesis, 1 μg of total RNA was reverse-transcribed using the Goldenstar™ RT6 cDNA Synthesis Kit (Tsingke, Beijing, China) with oligo(dT)_18_ primers in a 20 μL reaction. The resulting cDNA was diluted 10-fold with nuclease-free water. Subsequently, Real-time quantitative PCR (RT-qPCR) was performed using the 2 × T5 Fast qPCR Mix SYBR Green (Tsingke, Beijing, China) to detect the expression of mRNA, and *ALG9* was used as a housekeeping gene. The relative expression levels of individual genes were calculated by the 2^−∆∆Ct^ method [24]. All primers used in RT-qPCR are listed in Table S2.

### 2.7. Evaluation of Stress Tolerance

Stress tolerance of the engineered strains BY4741 *PGK1p-FLO1*, BY4741 *TPS1p-FLO1*, as well as the control strain BY4741, was evaluated using spot assays. Yeast strains were pre-cultured in YPD medium at 30 °C and 150 rpm for 12 h, followed by subculturing in fresh YPD medium for 10 h. Cells were harvested by centrifugation at 3000× *g* for 2 min and washed twice with sterile distilled water. For flocculating strains, cells were deflocculated using sterile 0.1 M sodium citrate buffer (pH 4.5). Cell cultures were adjusted to an OD_600_ of 1.0. Serial 10-fold dilutions were prepared, and 2 μL of the suspensions were spotted onto YPD agar plates containing 5 g/L acetic acid, 2 g/L furfural, 1.5 M NaCl, 2 mM sorbic acid, 40 mM propionic acid or 5 mM H_2_O_2_, respectively. The plates were then incubated at 30 °C. Besides that, osmotic stress tolerance was tested using plates containing 25% (*w*/*v*) glucose. For thermal tolerance assessment, plates were incubated at 42 °C, while plates without any inhibitory compounds at 30 °C were used as a control. All plates were incubated for 12–60 h according to the growth status. All experiments were performed in triplicate.

### 2.8. Analysis of Antioxidant Capacity and Intracellular ATP Concentration

Intracellular ATP levels and antioxidant capacity parameters including the ratio of reduced glutathione to oxidized glutathione ratio (GSH/GSSG), reactive oxygen species (ROS), and enzyme activities of catalase (CAT), superoxide dismutase (SOD) were measured using crude extracts of yeast cells. Specifically, yeast cells were harvested in the mid-logarithmic growth phase under 5 g/L acetic acid stress by centrifugation at 10,000× *g* for 2 min at 4 °C to rapidly separate cells from the medium. Cell pellets were washed twice with PBS buffer (pH 7.0) and resuspended in either PBS or lysis buffer provided with the assay kits. After that, crude extracts were then prepared by violent vortex with 0.5 mm glass beads for 60 s and then cooled in ice for 60 s between two vortexing. After repeating for 6 times, cell extracts were collected by centrifugation at 6000× *g* for 15 min at 4 °C. The resulting supernatants were used for subsequent analyses. Intracellular ATP concentration, ROS levels, GSH/GSSG ratio, CAT and SOD activities were determined by corresponding assay kits (Beyotime, Nantong, China) following the manufacturer’s instructions. The protein concentrations in the extracts were quantified with a BCA Protein Assay Kit (Vazyme Biotechnology, Nanjing, China). Three independent biological replicates were performed for each assay.

### 2.9. Statistical Analysis

Statistical analyses were performed on three independent biological replicates for all experiments, including intracellular ATP determination, enzyme activity assays, ethanol fermentation, and real-time quantitative PCR. The results were shown as means with standard deviations (SD). Data normality was assessed using the Shapiro–Wilk test prior to statistical analysis. Student’s *t*-test was used for statistical analyses with a significant level of *p*-value < 0.05 *.

## 3. Results and Discussion

### 3.1. Enhancement of Cell Flocculation by Promoter Replacement

It has been demonstrated that flocculation enhances the tolerance of *S. cerevisiae* to various environmental stresses, including low temperature, acetic acid, ethanol, furfural, and hydrogen peroxide [14,15,16,25,26]. However, this effect depends on floc size, which has a significant impact on cell growth, mass transfer, and fermentation performance [16,17]. As shown in our previous studies, 300 μm floc populations exhibited higher viability than 200 μm and 100 μm flocs under the 20% (*v*/*v*) ethanol shock. In that study, different floc size distributions were obtained by adjusting the stirring rates of the bioreactor [16]. To further investigate the influence of flocculation degree on stress tolerance, especially ethanol fermentation performance under acetic acid tolerance, and to avoid the potential effects caused by differences in rotational speed, we genetically modified the non-flocculating laboratory strain *S. cerevisiae* BY4741 to construct stable strains with varying flocculation capacities.

To enable *FLO1* expression in BY4741, its native promoter was replaced to eliminate Tup1-Cyc8 repression. The selection of promoters has a critical impact on optimizing gene expression and phenotype of *S. cerevisiae* [27,28]. Desirable flocculation intensity of the industrial yeast *S. cerevisiae* 4126 could be induced by expressing the *FLO1* gene under the control of constitutive 3-phosphoglycerate kinase (*PGK1*) promoter or the trehalose-6-phosphate synthase 1 (*TPS1*) promoter [29]. However, the effect of *FLO1* gene expression driven by these two promoters on laboratory yeast flocculation and associated stress tolerance has not yet been investigated, since promoter function can be influenced by the genetic background of the yeast strains [27]. In our study, the *FLO1* promoter in BY4741 was replaced with either the constitutive strong promoter *PGK1p* or the ethanol-inducible promoter *TPS1p*, generating recombinant strains BY4741 *PGK1p-FLO1* and BY4741 *TPS1p-FLO1*, respectively.

**Figure 1:**
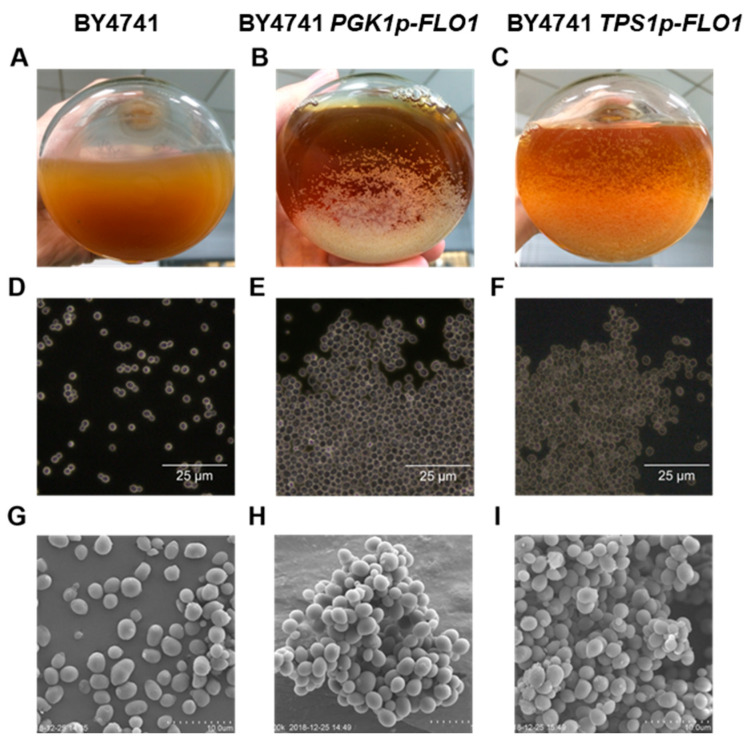
Flocculation phenotype observation of parent strain BY4741 and *FLO1* native promoter substitution strains BY4741 *PGK1p-FLO1* and BY4741 *TPS1p-FLO1*. (**A**–**C**), visual observation. (**D**–**F**), optical microscope. (**G**–**I**), scanning electron microscope. The scale bar represents 10 µm. (**A**,**D**,**G**), BY4741. (**B**,**E**,**H**), BY4741 *PGK1p-FLO1*. (**C**,**F**,**I**), BY4741 *TPS1p-FLO1*.

As illustrated in Figure 1, both promoter-replaced strains exhibited flocculation ability. Cells of BY4741 *PGK1p-FLO1* and BY4741 *TPS1p-FLO1* aggregated to form large particles. However, the two genetically modified strains showed significantly different flocculation degrees. BY4741 *PGK1p-FLO1* formed more compact and larger flocs, whereas BY4741 *TPS1p-FLO1* generated looser and smaller aggregates. This difference in floc morphology aligns with the known relative strengths of the *PGK1p* and *TPS1p* promoters. However, subsequent detection using high-resolution optical and scanning electron microscopy revealed no obvious differences in intercellular compactness between the two recombinant strains at the microscopic scale (Figure 1). These results indicate that the observed divergence in floc size and density is not attributable to the strength of cell–cell adhesion at the microscopic level. Instead, it may be governed by other factors influenced by promoter strength, such as the abundance of Flo1p adhesin displayed on the cell surface or the timing and rate of floc initiation. Based on initial visual and microscopic observations of floc morphology (Figure 1), strains expressing *FLO1* under the promoters *PGK1p* and *TPS1p* were preliminarily classified as strong flocculation and moderate flocculation, respectively. This phenotypic classification was subsequently confirmed by quantitative measurements of flocculation rate and particle size distribution, as detailed in the following section.

### 3.2. Variation in Flocculation Character by Different Recombinants

To further quantitatively evaluate flocculation states resulting from differential *FLO1* expression, flocculation rates were initially determined. As shown in Figure S1, the control strain BY4741 exhibited no flocculation ability, with a flocculation rate of 6.2% after 5 min, attributable to natural sedimentation. In contrast, nearly all cells of BY4741 *PGK1p-FLO1* and BY4741 *TPS1p-FLO1* sedimented rapidly after undergoing the process of deflocculation–reflocculation followed by 5-min settling, consistent with the behavior of the strongly flocculating yeast SPSC01. Quantitative analysis confirmed flocculation rates approaching 100% for both recombinant strains, which suggested that both engineered strains demonstrated robust flocculation ability in the absence of any stress. These results also indicate that although flocculation rate effectively distinguishes the recombinants from the non-flocculating parent BY4741, it is not a suitable parameter for differentiating their relative flocculation strengths.

**Figure 2:**
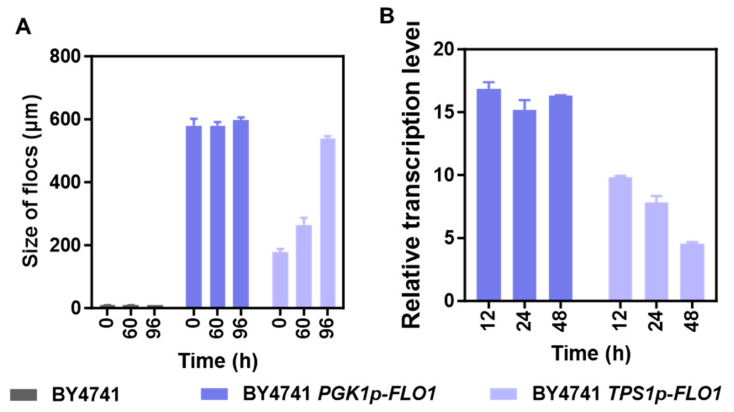
Dynamics of floc size and *FLO1* transcription in recombinant strains under acetic acid stress. (**A**) Detection of flocs size distribution of BY4741, BY4741 *PGK1p-FLO1*, and BY4741 *TPS1p-FLO1* using FBRM; (**B**) Transcriptional levels of *FLO1* in BY4741 *PGK1p-FLO1* and BY4741 *TPS1p-FLO1* relative to the control strain BY4741 at different fermentation time points. *ALG9* was used as a reference gene.

Subsequently, floc particle diameter of these yeast strains during fermentation under acetic acid stress was monitored by Focused Beam Reflectance Measurement (FBRM). Samples taken at inoculation (0 h), mid-fermentation (60 h), and terminal fermentation (96 h) revealed distinct floc formation patterns. Both BY4741 and BY4741 *PGK1p-FLO1* maintained consistent particle sizes throughout fermentation (Figure 2A). The measurement of BY4741 was maintained at 9.6 μm, corresponding to single-cell diameter, while BY4741 *PGK1p-FLO1* formed stable flocs averaging 585 μm at all sampling points, indicating growth phase and fermentation stage independence. Conversely, BY4741 *TPS1p-FLO1* exhibited a progressive increase in mean floc diameter from 178 μm (0 h) to 265 μm (60 h), ultimately reaching 539 μm (96 h). Notably, BY4741 *TPS1p-FLO1* flocs reached dimensions comparable to BY4741 *PGK1p-FLO1* at the final point. It indicates that the classification of strong versus moderate flocculation primarily reflects the kinetics and stability of floc formation rather than the absolute floc size achieved under prolonged fermentation. In addition, this dynamic size change suggested a potential link to *FLO1* expression. We then investigated the transcription levels of *FLO1* in the cells of BY4741 *PGK1p-FLO1* and BY4741 *TPS1p-FLO1* relative to BY4741. As illustrated in Figure 2B, differential expression patterns of *FLO1* were found in the two genetically modified flocculating strains. BY4741 *PGK1p-FLO1* maintained consistently high *FLO1* expression level, whereas BY4741 *TPS1p-FLO1* showed progressively declining transcript levels in the fermentation process. Specifically, the relative transcript levels of *FLO1* were 9.8, 7.8, and 4.5 at 12 h, 24 h, and 48 h, respectively. The high expression of *FLO1* in the cells of BY4741 *PGK1p-FLO1* was consistent with its strong flocculation and stable large flocs. However, it is worth noting that compared to ZLH01, an industrial *S. cerevisiae* 4126 derivative with *FLO1* expression driven by the *TPS1* promoter [29], BY4741 *TPS1p-FLO1* displayed progressively enhanced flocculation during fermentation. However, ZLH01 primarily showed increased flocculation rate [29], while BY4741 *TPS1p-FLO1* maintained a stable flocculation rate but exhibited increasing floc diameter (Figure 2A). Furthermore, transcriptional level of *FLO1* in ZLH01 showed sustained upregulation dependent on ethanol concentration [29], contrasting with the continuous downregulation observed in BY4741 *TPS1p-FLO1* at all sampling points. The above-mentioned discrepancies may arise from differences in genetic background, sampling times, fermentation conditions, and crucially, acetic acid stress. The stress condition likely alters yeast physiological characteristics, potentially affecting the response of *TPS1p* to ethanol. This potential cross-talk highlights a critical caveat when employing environmentally sensitive promoters in complex industrial media, where multiple inhibitors are present. In addition, it suggests that it is important to validate promoter behavior under industrially relevant conditions when designing robust yeast strains.

### 3.3. Effects of Flocculation Degree on Stress Tolerance of S. cerevisiae

**Figure 3:**
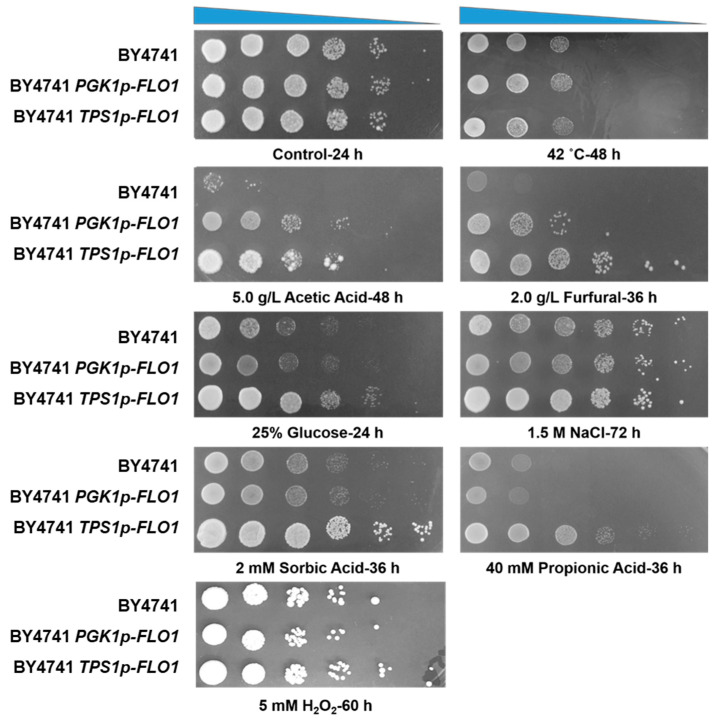
Spotting assay of BY4741, BY4741 *PGK1p-FLO1*, and BY4741 *TPS1p-FLO1* under various environmental stresses. Serial 10-fold dilutions (from 10^0^ to 10^−5^) of each strain were spotted onto YPD agar plates containing one of the following inhibitors: 5.0 g/L acetic acid, 5.0 g/L furfural, 1.5 M NaCl, 2 mM sorbic acid, 40 mM propionic acid or 5 mM H_2_O_2_, with a YPD plate without any inhibitors served as the control. For osmotic stress assay, the test was performed using YPD medium containing 250 g/L glucose. Except for thermal tolerance detection at 42 °C, other plates were incubated at 30 °C. All experiments were performed in triplicate with consistent results.

**Figure 4:**
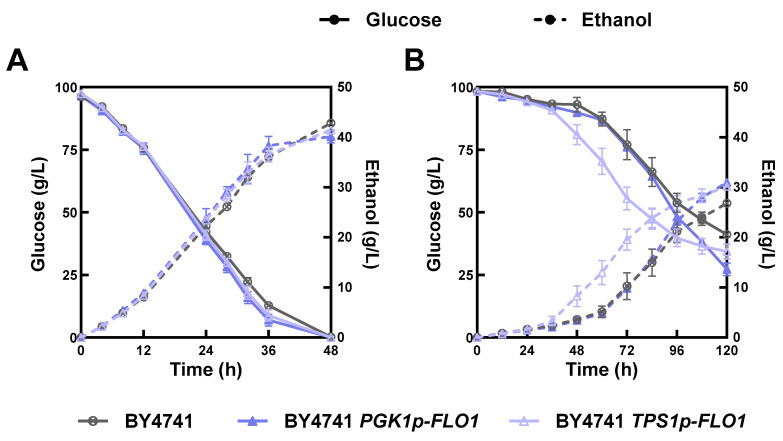
Ethanol fermentation performance of BY4741, BY4741 *PGK1p-FLO1*, and BY4741 *TPS1p-FLO1* under different conditions. Batch fermentation was performed at 30 °C, 150 rpm and aerobic condition in 250 mL Erlenmeyer flasks containing 4.0 g/L yeast extract, 3.0 g/L peptone and 100.0 g/L glucose under stress-free condition (**A**) or 5.0 g/L acetic acid addition (**B**).

Previous studies indicate that flocculation degree influences yeast tolerance to inhibitors like furfural and ethanol [15,16]. To comprehensively evaluate whether flocculation modulates multiple stress responses and the effect of flocculation degree, growth ability of non-flocculating (BY4741), strong-flocculating (BY4741 *PGK1p-FLO1*), and moderate-flocculating (BY4741 *TPS1p-FLO1*) strains under diverse stress conditions was assessed using spot assays (Figure 3) and ethanol fermentation (Figure 4).

Spot assays revealed that flocculation did not affect growth under stress-free conditions. When under the stress of 5.0 g/L acetic acid or 2.0 g/L furfural, both flocculating strains exhibited significantly improved tolerance compared to BY4741. Notably, BY4741 *TPS1p-FLO1* demonstrated superior growth to BY4741 *PGK1p-FLO1* in the presence of these inhibitors (Figure 3). Acetic acid and furfural are main inhibitors released during lignocellulose pretreatment and severely impair fermentation performance through affecting cell growth and ethanol productivity [6,30]. The superior performance of the flocculating strains, particularly BY4741 *TPS1p-FLO1*, suggested that this strategy contributed to construct robust strains in lignocellulose-based biofuels and biochemicals production. Moreover, BY4741 *TPS1p-FLO1* showed enhanced growth under preservative stress such as 2 mM sorbic acid or 40 mM propionic acid, and osmotic stress (25% glucose), while BY4741 *PGK1p-FLO1* performed similarly to BY4741. In contrast, no significant growth differences were observed among strains under NaCl stress (1.5 M), oxidative stress (5 mM H_2_O_2_), or thermal stress (42 °C). The higher tolerance to 25% glucose indicted that BY4741 *TPS1p-FLO1* was conducive to high-gravity fermentation. In large-scale industrial fermentation, high-gravity fermentation has been a key strategy for increasing production capacity and reducing costs [31]. Additionally, acetic acid, sorbic acid and propionic acid are widely used as preservatives in various beverages and food products [32]. Tolerance to food preservatives enhances industrial yeast’s robustness by allowing it to outperform contaminants and ferment non-sterile substrates. On the other hand, although acetic acid stress induced ROS accumulation [8], no obvious difference in H_2_O_2_ tolerance of flocculating yeast strains relative to the control strain was observed. According to our previous studies, flocculation endowed the industrial strain SPSC01 with improved acetic acid stress tolerance but weakened H_2_O_2_ tolerance. For the mutants derived from laboratory yeast strain CEN.PK 113-7D, flocculation properties enhanced its tolerance to furfural but had no effect on acetic acid stress [15]. Collectively, these results demonstrate that flocculation effects are strain-dependent and influenced by flocculation degree. It is important to note that for this spotting assay, cells of the flocculating strains BY4741 *PGK1p-FLO1* and BY4741 *TPS1p-FLO1* were tested in a deflocculated state. Therefore, the enhanced growth observed here primarily reflects the inherent physiological tolerance conferred to individual cells by the genetic modifications, rather than the direct physical protection offered by the multicellular floc structure.

To further investigate the acetic acid tolerance of these strains with different degree of flocculation, ethanol fermentation was performed in the presence of 5.0 g/L acetic acid. Flocculation did not affect ethanol production without acetic acid stress (Figure 4A). Contrastingly, BY4741 *TPS1p-FLO1* exhibited a shorter lag phase (36 h) than BY4741 and BY4741 *PGK1p-FLO1* (48 h) under the stress of acetic acid. This result implied the advantage of flocculation on cell growth protection to acetic acid. As shown in Figure 4B, residual glucose of BY4741 *PGK1p-FLO1* and BY4741 *TPS1p-FLO1* were 27.3 g/L and 34.4 g/L, whereas there was 41.2 g/L residual glucose for the control strain after 120 h fermentation. Furthermore, obvious improvement in ethanol production by *FLO1* overexpression was observed. The control strain BY4741 produced 26.8 g/L of ethanol, while the final concentrations of ethanol production were 31.0 g/L and 30.2 g/L by strains BY4741 *PGK1p-FLO1* and BY4741 *TPS1p-FLO1*, which were 15.6% and 12.6% higher than that of BY4741, respectively. Interestingly, BY4741 *PGK1p-FLO1* showed rapid glucose consumption during late fermentation, whereas glucose consumption rate of BY4741 was severely inhibited after fermentation for 84 h. We speculate that this lower activity of BY4741, in contrast to the sustained metabolism of the flocculating strains, may be attributed to a loss of cell viability under prolonged acetic acid stress. These results indicate that flocculation enhances acetic acid tolerance, with moderate flocculation (BY4741 *TPS1p-FLO1*) potentially optimizing the balance between growth and fermentation performance. This aligns with our previous studies that ethanol tolerance of floc populations was increased with the average size distributed from 100 to 400 μm. When the average floc size further increased to 400 μm, decreased ethanol tolerance was recorded [16].

### 3.4. Enhanced Expression of Stress-Responsive Genes in Flocculating Yeast

As reported, acetic acid stress induces oxidative damage and intracellular reactive oxygen species (ROS) accumulation [33], while enhanced total antioxidant capacity facilitates scavenging of excessive ROS. Robust acetic acid-tolerant strains have been shown to exhibit lower ROS levels [34,35,36,37]. In this study, BY4741 *TPS1p-FLO1* exhibited obviously higher tolerance to 5 g/L acetic acid than that of BY4741 *PGK1p-FLO1* and the control strain BY4741 in spotting assay and early stage of batch fermentation. To investigate whether flocculating yeast enhances acetic acid tolerance through elevated antioxidative activity and the difference between flocculating yeast strains, we measured transcriptional levels of several key stress-responsive genes during exposure to 5 g/L acetic acid, including *CTT1*, *CTA1*, *HAA1*, *SOD1*, *MSN2* and *HSP12*. Meanwhile, antioxidant capacity and intracellular ROS levels of BY4741, BY4741 *PGK1p-FLO1*, and BY4741 *TPS1p-FLO1* under 5 g/L acetic acid stress were detected.

**Figure 5:**
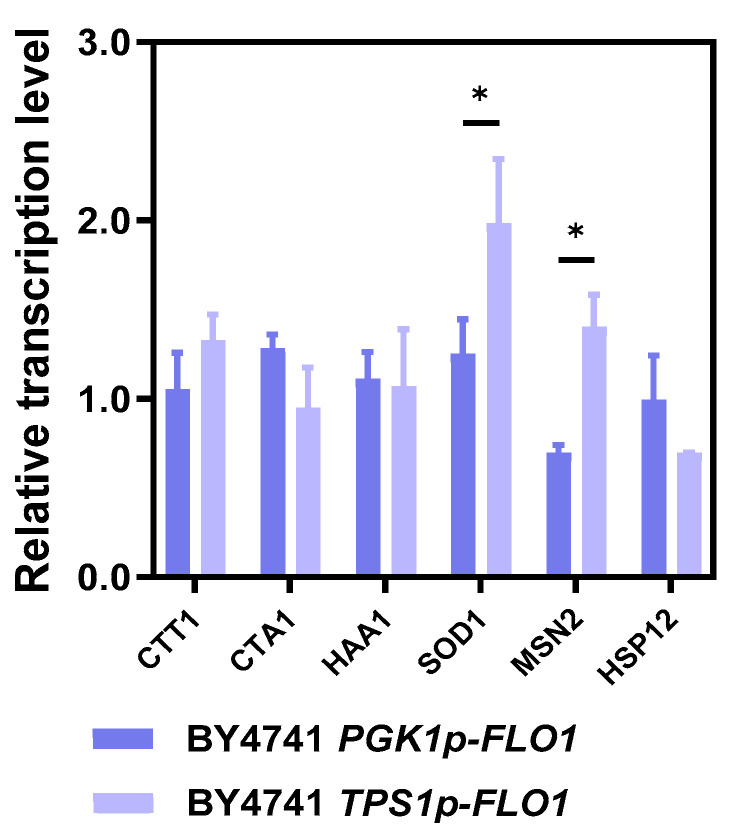
Relative transcription level of stress responsive genes in BY4741 *PGK1p-FLO1* and BY4741 *TPS1p-FLO1* compared to the wild-type strain BY4741 under the stress of acetic acid. The yeast strains were grown in YPD100 supplemented with 5 g/L acetic acid and total RNA was extracted from cells at logarithmic growth phase (36 h). *ALG9* was used as a reference gene. “*” represents *p*-value < 0.05.

According to the results in Figure 5, the moderate-flocculating strain BY4741 *TPS1p-FLO1* exhibited obvious upregulation of *SOD1* and *MSN2* relative to that of the wild-type strain by 1.98- and 1.40-fold, which encodes superoxide dismutase and a vital stress-responsive transcription factor, respectively. To be more specific, *SOD1* encodes a crucial component of the enzymatic antioxidant defense system. Enhanced SOD activity modulates ROS accumulation and improves oxidative stress resistance in acetic acid-tolerant strains [8,38]. Significant SOD activity increases were previously observed in wild-type strain GRF18U after acetic acid exposure [39], and elevated *SOD1* expression occurs in engineered strains with higher acetic acid tolerance such as mutants with *RTT109* deletion and purine de novo synthesis genes overexpression [40,41]. This suggests that *SOD1* upregulation acts as a defensive response to oxidative stress. Msn2, regulated by phosphorylation status, translocates to the nucleus under stress to activate multiple stress-defense genes [42,43]. For instance, Msn2 mediates *CTT1* regulation through the Hog1 pathway during acetic acid stress [44]. Furthermore, *SOD1* and *MSN2* in BY4741 *TPS1p-FLO1* also showed significantly higher transcription level compared to BY4741 *PGK1p-FLO1*. Therefore, the transcriptional changes in *SOD1* and *MSN2* may contribute to enhanced acetic acid tolerance of BY4741 *TPS1p-FLO1*.

Subsequently, antioxidant capacity was determined. However, even though the expression of *SOD1* was upregulated in BY4741 *TPS1p-FLO1* compared to BY4741 *PGK1p-FLO1* and BY4741, no significant differences were observed in superoxide dismutase (SOD) activity among the three strains during the early fermentation stage (12 h) (Figure S2). The result suggested that the flocculation state might influence the translation, stabilization or degradation of superoxide dismutase under the stress of acetic acid. Besides that, catalase (CAT) activity, which is generally related to decreased ROS accumulation in acetic acid tolerant strain [12], was also no difference among these three yeast strains (Figure S2). Similarly, there was also no variations in CAT, SOD activities or reduced glutathione/oxidized glutathione ratio (GSH/GSSG) when detecting the cells after fermentation for 24 h (Figure S3). Consistent with these observations, intracellular ROS levels remained comparable across all strains at 24 h (Figure S3A), and no obvious differences in growth in the presence of 5 mM H_2_O_2_ were observed among these strains (Figure 3). These results suggest that, while transcriptional changes in oxidative stress-related genes occurred, the overall antioxidant enzyme activities and redox homeostasis were effectively maintained in all strains under the tested conditions. Thus, the enhanced acetic acid tolerance conferred by promoter-driven flocculation does not appear to be primarily mediated through alleviation of oxidative stress, but may instead involves other mechanisms.

### 3.5. Flocculation Enhances Acetic Acid Tolerance by Sustaining High Intracellular ATP Levels

A key mechanism of acetic acid toxicity is the reduction in intracellular ATP levels. Entry of 1 M acetic acid consumes approximately 1 M ATP. Under the stress of 160 mM acetic acid, *S. cerevisiae* exhibited a 30% decrease in intracellular ATP content [45]. Moreover, under stress conditions inducing cytosolic acidification, the H^+^-ATPase proton pump (Pma1) is essential for maintaining cytoplasmic pH homeostasis by pumping out excess protons, which is an ATP-dependent process [46]. Acetic acid tolerance can also be enhanced by acetate conversion to acetyl-CoA via acetyl-CoA synthetases (Acs1 and Acs2). Although *ACS2* overexpression confers higher acetic acid resistance, it consumes one ATP per acetyl-CoA formed [47]. In addition, ATP is required for expressing stress-response genes and modifying regulatory pathway proteins. Therefore, maintaining stable intracellular ATP levels is critical for yeast acetic acid detoxification [48].

**Figure 6:**
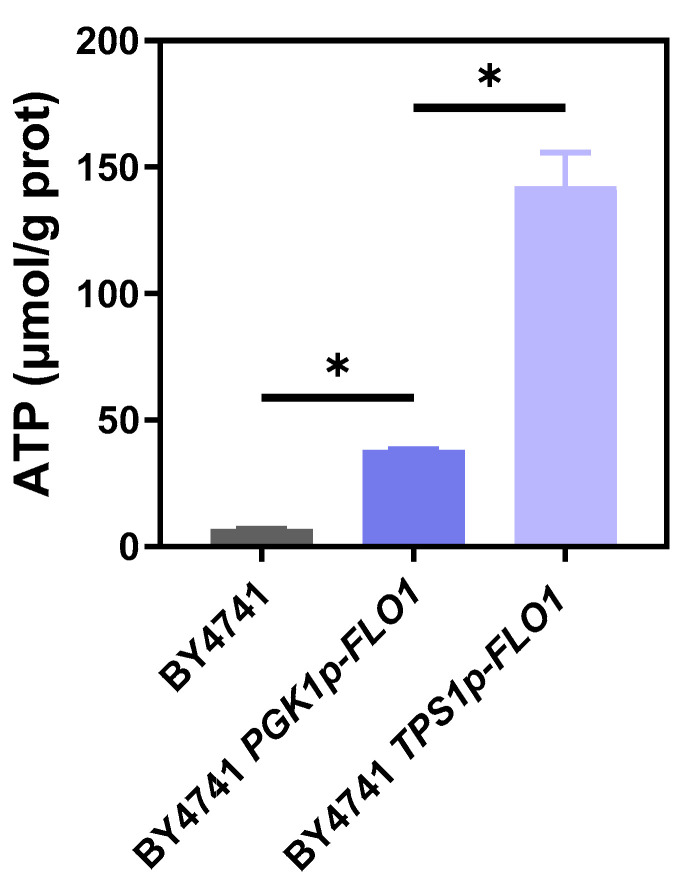
The concentration of intracellular ATP in BY4741, BY4741 *PGK1p-FLO1*, and BY4741 *TPS1p-FLO1* under 5.0 g/L acetic acid. The yeast cells were harvested at exponential growth phase for triplicate analysis. “*” represents *p*-value < 0.05.

Considering that energy production is closely related to acetic acid tolerance, we then measured the difference in intracellular ATP levels between the two flocculating yeast strains and the non-flocculating control strain BY4741 under 5 g/L acetic acid stress. As shown in Figure 6, ATP content of BY4741 was 6.9 nmol/mg protein, while the ATP production of 38.3 and 142.2 nmol/mg protein was detected in BY4741 *PGK1p-FLO1* and BY4741 *TPS1p-FLO1*, which was significantly higher than that of BY4741. These results indicate that, specifically under acetic acid stress, flocculating strains were capable of maintaining intracellular ATP at levels significantly higher than that in the non-flocculating control. Notably, the moderately flocculating strain BY4741 *TPS1p-FLO1* displayed even higher ATP levels than the strongly flocculating strain BY4741 *PGK1p-FLO1*. This observation implies that moderate flocculation might endure reduced mass transfer resistance, conferring cells an advantage in utilizing carbon and nitrogen sources and in absorbing oxygen. Consequently, these cells can generate more ATP to compensate for the energy deficit induced by acetic acid stress. Supporting this notion, studies from our group showed that ethanol tolerance and plasma membrane ATPase activity improved with floc particle size increased from 100 μm to 300 μm, whereas its performance was decreased when the particle size was up to 400 μm [16]. Therefore, we propose that by enhancing acetic acid tolerance, flocculation reduced cellular ATP expenditure, thus resulting in enhanced cell performance and fermentation capacity. Our ATP measurement represent a population average value, and spatial heterogeneity was not directly assessed. We speculate that exterior cells endure higher stress but benefit from better nutrient consumption, whereas interior cells are partially protected by exterior cells. This population-wide enhancement of energetic capacity indicted that flocculation may serve as a multicellular strategy to sustain ATP-demanding stress responses under inhibitor pressure.

In the future study, to further elucidate the spatial heterogeneity and molecular mechanisms underlying flocculation-mediated stress protection, it would be highly valuable to dissect flocs and analyze gene expression profiles from exterior versus interior cells using single-cell tools such as single-cell RNA sequencing (scRNA-seq) [49] or spatial transcriptomics [50]. Such approaches could reveal whether cells located at different positions within a floc exhibit distinct transcriptional responses to acetic acid stress, particularly in terms of energy metabolism, detoxification pathways, and stress-responsive gene expression. This investigation would provide a more refined understanding of how multicellular aggregation distributes physiological burdens and synergistically enhances population-level tolerance, thereby offering new insights for designing precisely engineered flocculating yeasts tailored to specific inhibitor-rich environments.

### 3.6. Ethanol Fermentation in Bioreactors

**Figure 7:**
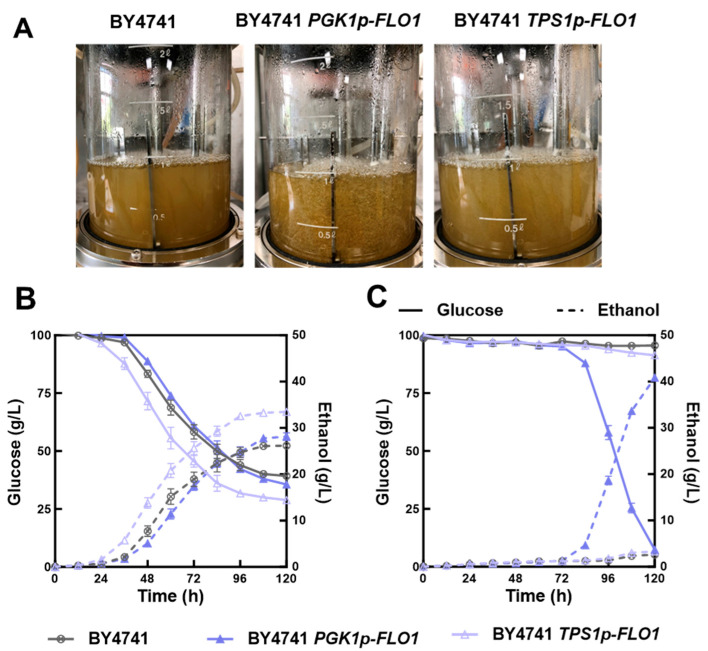
Comparison of flocculation phenotypes and ethanol fermentation performance between the parental strain BY4741 and recombinant strains in bioreactors. (**A**) The flocculation phenotype of BY4741, BY4741 *PGK1p-FLO1*, and BY4741 *TPS1p-FLO1* when fermenting in bioreactor with 5 g/L acetic acid; Ethanol fermentation performance of BY4741, BY4741 *PGK1p-FLO1*, and BY4741 *TPS1p-FLO1* in bioreactor under different conditions. (**B**) 5.0 g/L acetic acid (without pH adjustion). (**C**) 7.5 g/L acetic acid (pH 4.0).

Due to the limited volume of Erlenmeyer flasks, flocculation particles were unevenly distributed with significant size variability in the fermentation broth. To overcome this limitation and validate the stress tolerance under industrially relevant scalable conditions, ethanol fermentation performance under acetic acid stress was evaluated for the three strains in a larger-scale bioreactor. As illustrated in Figure 7A, the moderately flocculating strain BY4741 *TPS1p-FLO1* formed smaller, more uniform flocs in the bioreactor, whereas the strongly flocculating BY4741 *PGK1p-FLO1* generated larger, more heterogeneous aggregates. Additionally, cells of BY4741 *PGK1p-FLO1* exhibited a greater tendency to adhere to the impeller and sampling port compared to BY4741 *TPS1p-FLO1*, potentially impairing fermentation efficiency.

In lignocellulosic hydrolysates, the concentration of acetic acid generally ranges from 1 to 10 g/L [51]. In this study, ethanol fermentations were conducted under two stress conditions. The first condition employed 5.0 g/L acetic acid without pH adjustment, matching the stress intensity of the flask fermentations. To assess flocculation effectiveness under more severe stress, a second condition used 7.5 g/L acetic acid. To avoid poor growth of yeast cells, the pH maintained at 4.0 throughout the fermentation. As demonstrated in Figure 7B,C, BY4741 *TPS1p-FLO1* showed superior suitability for fermentation at 5.0 g/L acetic acid, while BY4741 *PGK1p-FLO1* exhibited advantages under the higher stress level (7.5 g/L). Consistent with flask fermentation results, BY4741 and BY4741 *PGK1p-FLO1* displayed similar glucose consumption and ethanol production rates at 5.0 g/L acetic acid, whereas BY4741 *TPS1p-FLO1* achieved better fermentation performance. However, all strains were severely inhibited under 7.5 g/L acetic acid supplementation. Notably, BY4741 *PGK1p-FLO1* resumed ethanol production rapidly after 72 h of fermentation. Conversely, growth and fermentation of BY4741 *TPS1p-FLO1* and BY4741 remained completely inhibited until 120 h. This suggests that only the strongly flocculating strain could endure such severe stress conditions.

Based on the fermentation performance in both flasks and the larger-scale bioreactors, we proved that enhanced flocculation by replacing the *FLO1* promoter was conducive to improve the acetic acid tolerance of strain BY4741. This indicates the applicability of flocculating yeast in scaled-up bioreactors, supporting the potential for industrial implementation of flocculation. Importantly, the optimal flocculation degree should match the inhibitor concentration present in lignocellulosic hydrolysates. Moderate flocculation appears optimal for balancing stress tolerance with cell growth and fermentation efficiency under mild stress, while strong flocculation is essential for cell survival and sustained ethanol production under severe stress conditions. However, it is worth noting that the activity of *TPS1p* might be influenced by other inducers present in lignocellulosic hydrolysates containing acetic acid, furfural, and phenolics. The behavior of such sensitive promoters may be unpredictable or strain-dependent, as shown in the differing expression profiles between BY4741 *TPS1p-FLO1* and ZLH01. Thus, inducible promoters are valuable for dynamic gene regulation, but their industrial application demands rigorous validation under actual fermentation conditions.

Intriguingly, a recent report shows that increased copy number of the transcriptional repressor *TUP1* is beneficial for enhanced ethanol tolerance [52]. Tup1p is a known repressor of *FLO1* expression, these findings suggest a potential trade-off in stress adaptation that elevated ethanol tolerance strain may inherently suppress flocculation and its associated benefits for acetic acid tolerance. Conversely, strategies that promote flocculation for acetic acid resistance, such as the *FLO1* overexpression employed here, might require an adjustment of the Tup1p regulatory network. This highlights the complex and sometimes opposing genetic demands imposed by different fermentation inhibitors. In our work, the flocculating strains maintained efficient ethanol production under acetic acid stress (Figure 4), indicating that the engineered *FLO1* expression did not impose a severe fitness cost in terms of ethanol tolerance under the tested conditions. However, the Tup1-Flo1 regulatory network likely represents a critical node for balancing adaptation to mixed-inhibitor environments. Future efforts to decouple this regulation or evolve strains under combined stresses may further optimize yeasts for complex industrial conditions.

## 4. Conclusions

This study demonstrates that fine-tuning flocculation intensity through *FLO1* promoter engineering is a powerful strategy for enhancing acetic acid tolerance in *S. cerevisiae*, and the effect was influenced by the degree of flocculation. Under mild stress caused by 5.0 g/L of acetic acid, the moderately flocculating strain BY4741 *TPS1p-FLO1* exhibited superior growth and fermentation efficiency. In contrast, the strongly flocculating BY4741 *PGK1p-FLO1* showed a survival advantage under more severe stress cause by 7.5 g/L acetic acid. Further studies suggested that the enhanced tolerance correlated with the ability of flocculating strains to maintain higher intracellular ATP levels under stress, which may support energy-demanding detoxification and homeostasis processes. These findings highlight the importance of balancing stress tolerance with metabolic performance by fine-tuning flocculation intensity. This work also provides a strategy for engineering robust industrial yeast strains to facilitate efficient lignocellulosic biorefinery processes under inhibitor-rich conditions.

## Figures and Tables

**Table 1:** Strains and plasmids used in this study.

Strains	Description	Source
*Escherichia coli* DH5α	For plasmid construction and propagation	Lab preservation, Shanghai, China
BY4741	*S. cerevisiae*, haploid, *MATa*, *his3*∆*1*, *leu2*∆*0*, *met15*∆*0* and *ura3*∆*0*	Euroscarf, Oberursel, Germany
Cas9-G418	p414, ARS/CEN, *KanMX*, *TEF1p-Sp*Cas9-*CYC1t*	Lab preservation, Shanghai, China
BY4741-Cas9	Transform Cas9-G418 plasmid into BY4741	This study
gRNA-*FLO1p*	Plasmid expressing gRNA targeting to the native promoter of *FLO1*	This study
BY4741 *PGK1p-FLO1*	BY4741, *FLO1p*::*PGK1p*	This study
BY4741 *TPS1p-FLO1*	BY4741, *FLO1p*::*TPS1p*	This study

## Data Availability

All data generated or analyzed during this study are included in this published article and its Supplementary Information Files.

## Supplementary Materials

The following supporting information can be downloaded at: https://www.mdpi.com/article/10.3390/jof12010047/s1, Table S1. Primers used in this study; Table S2. Primers used in RT-qPCR analysis; Figure S1. Flocculation rate of parent strain BY4741 and *FLO1* native promoter substitution strains BY4741 *PGK1p-FLO1* and BY4741 *TPS1p-FLO1*; Figure S2. Antioxidant capacity of BY4741, BY4741 *PGK1p-FLO1* and BY4741 *TPS1p-FLO1* under 5.0 g/L acetic acid stress (12 h); Figure S3. Antioxidant capacity of BY4741, BY4741 *PGK1p-FLO1* and BY4741 *TPS1p-FLO1* under 5.0 g/L acetic acid stress (24 h).
