# Supplementary material for: Tuning FLO1 Expression via Promoter Engineering Modulates Flocculation Degree and Acetic Acid Stress Tolerance in Saccharomyces cerevisiae
Source: J Fungi (Basel). 2026 Jan 9;12(1):47. doi: 10.3390/jof12010047 (PMC12843017; doi:10.3390/jof12010047)
Supplement: Supplementary file 1 [file jof-12-00047-s001.zip › jof-4022949-supplementary.pdf]

Table S1. Primers used in this study

**Primers**	**Fragments (5′→3′)**	**Description**
v-TEF1p-F	CTCTTCCAGATTTTCTCGGA	Verify the existence of Cas9 plasmid and the substitution of native promoter by *TEF1p*
v-Cas9-R	GATATTGCCAAAGATTGGGTG	Verify the existence of Cas9 plasmid
gRNA-FLO1p-F	GATCGTTTGATGTAAGCTCTCTTC	Anneal to obtain gRNA fragment for being fused to gRNA_clone plasmid backbone
gRNA-FLO1p-R	AAACAAGAGAGCTTACATCAAAC	
Donor- PGK1pFLO1-F	CTTCCAGTATGCTTTCACGGAATTATTTCTCATGTACATTTAGCTCCATTTCCAGTGCCTGAAGTACCTTCAAAGAATGG	Amplify donor DNA (*PGK1p*) with homologous arms for substituting native *FLO1* promoter
Donor-PGK1pFLO1-R	ACTAGTTAGTGCCAGAAGTGTAAAGACTGCCAAAAACATATAGCGATGAGGCATTGTCATTTGTTTTATATTTGTTGTAAAAAG	
Donor-TPS1pFLO1-F	CTTCCAGTATGCTTTCACGGAATTATTTCTCATGTACATTTAGCTCCATTTCCAGTGCCTCAACCCGGTCTCGAAGAACA	Amplify donor DNA (*TPS1p*) with homologous arms for substituting native *FLO1* promoter
Donor-TPS1pFLO1-R	ACTAGTTAGTGCCAGAAGTGTAAAGACTGCCAAAAACATATAGCGATGAGGCATTGTCATAGTTCTATGTCTTAATAAGTC	
v-PGK1p-F	CGATTTGGGCGCGAATCCTT	Verify the substitution of native *FLO1* promoter by *PGK1p* or *TPS1p*
v-TPS1p-F	GGTGATAGCCATATCTTCG	
v-FLO1-R	ACTAGTTAGTGCCAGAAGTG	

Table S2. Primers used in RT-qPCR analysis

**Primers**	**Sequence (5′→3′)**
RT-*CTT1*-F	CAGTCCAACGAACACTTGTA
RT-*CTT1*-R	GCAATTGCTGATAGTTGGCT
RT-*CTA1*-F	TGCCAACTACCGTAGTATGC
RT-*CTA1*-R	GTAGCCTCTTCTATGGTCAA
RT-*HAA1*-F	AATCCTGAAGGTGTTTGCAC
RT-*HAA1*-R	GCATGATATTTGCAAACCTC
RT-*SOD1*-F	GGTGTTGTCAAGTTCGAACA
RT-*SOD1*-R	AGCAGAGACACAACCATTGG
RT-*MSN2*-F	CGAAACTAATTTATCGCCTC
RT-*MSN2*-R	GGGTTAGTGTCAATGAAAAG
RT-*HSP12*-F	GGATTCGGTGAAAAAGCTTC
RT-*HSP12*-R	AAGACACCCTTGTTGTCTTC

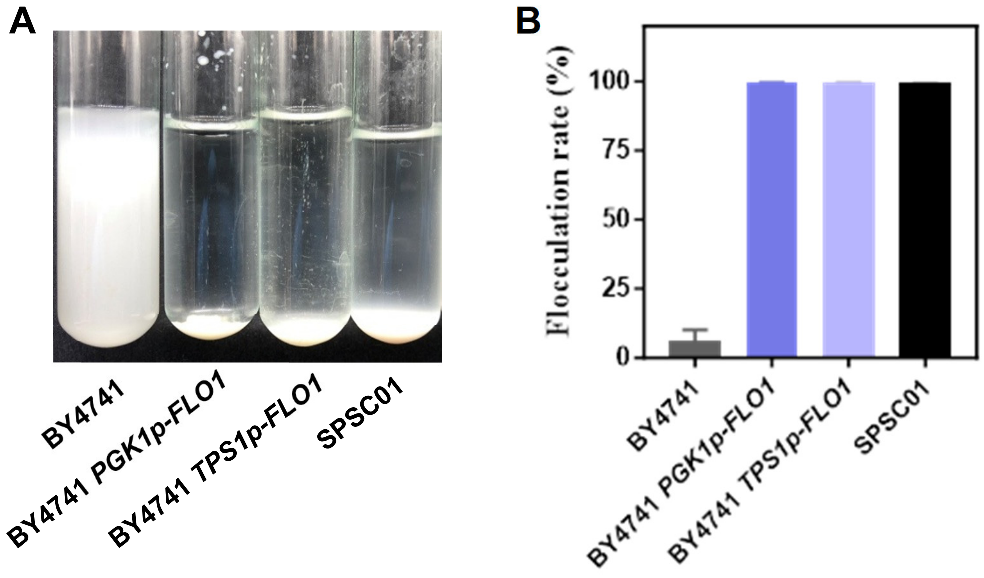

Figure S1. Flocculation rate of parent strain BY4741 and *FLO1* native promoter substitution strains BY4741 *PGK1p-FLO1* and BY4741 *TPS1p-FLO1*. A, Subsidence phenotype of *S. cerevisiae* strains after 5 min standing when detecting flocculation rate. B, Flocculation rate of S. cerevisiae strains.

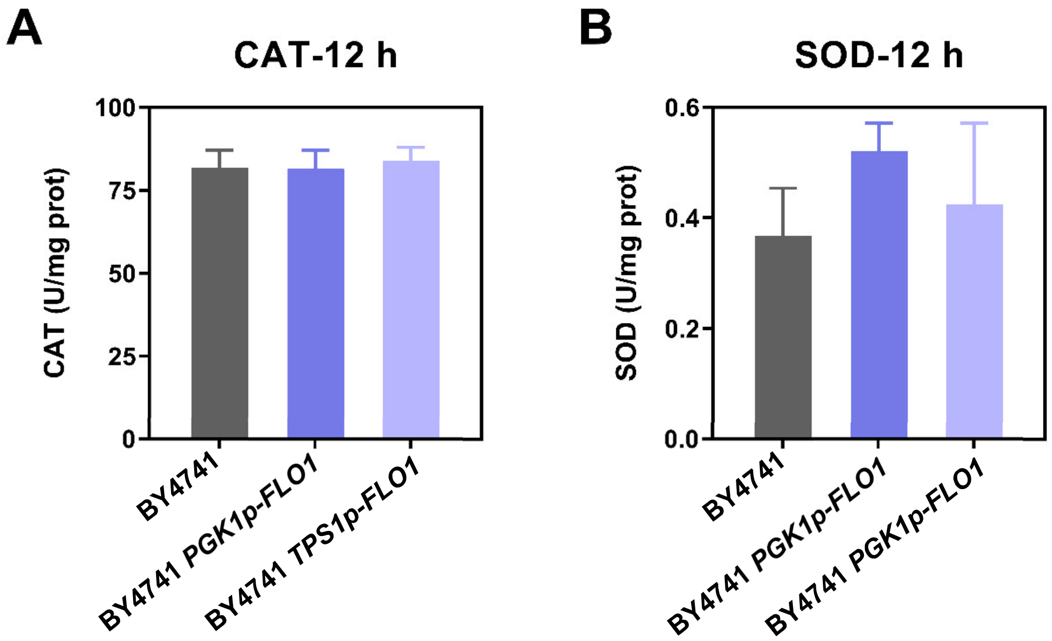

Figure S2 Antioxidant capacity of BY4741, BY4741 *PGK1p-FLO1* and BY4741 *TPS1p-FLO1* under 5.0 g/L acetic acid stress (12 h). A, the activity of catalase (CAT). B. the activity of superoxide dismutase (SOD).

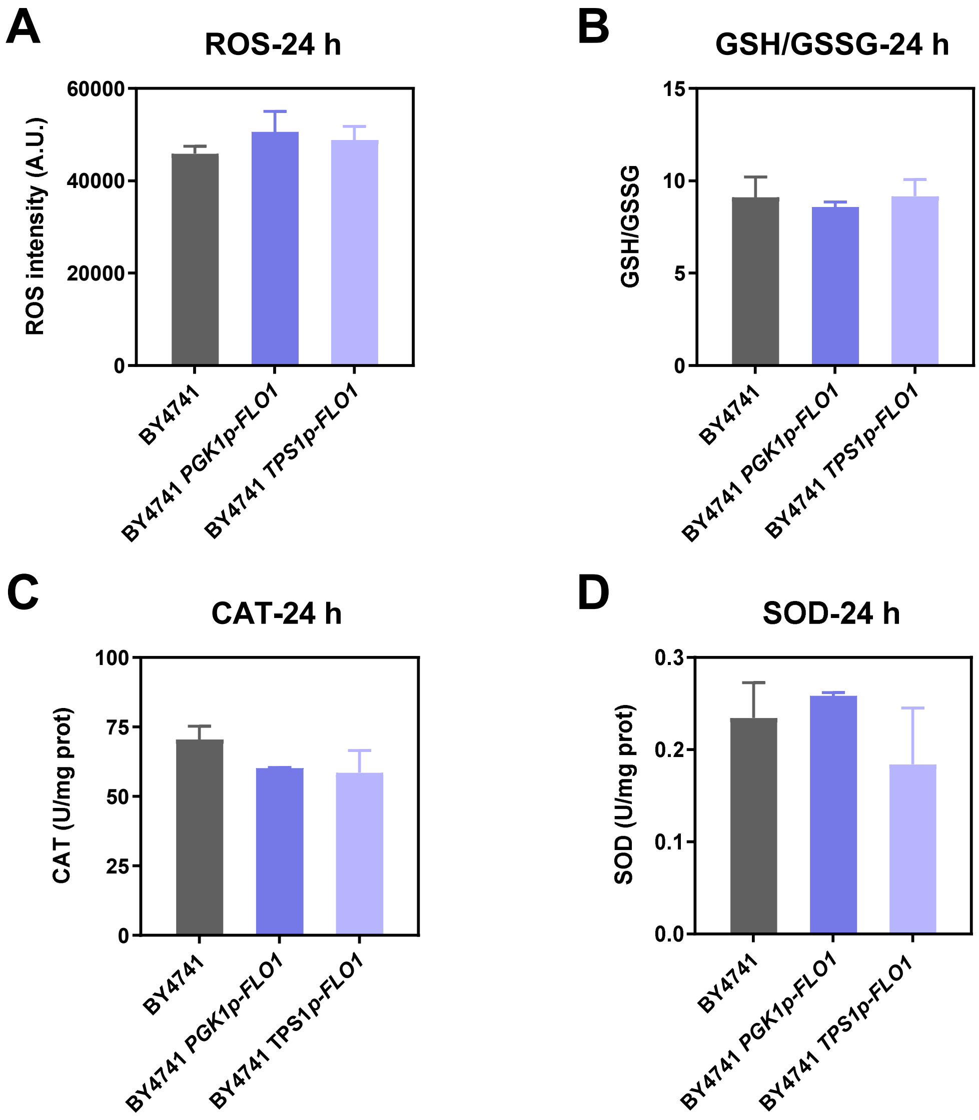

Figure S3 Antioxidant capacity of BY4741, BY4741 *PGK1p-FLO1* and BY4741 *TPS1p-FLO1* under 5.0 g/L acetic acid stress (24 h). A, intracellular ROS level. A.U. represents arbitrary unit. B, the ratio of reduced glutathione (GSH) to oxidized glutathione (GSSG). C, the activity of catalase (CAT). D. the activity of superoxide dismutase (SOD).
